# Gamifying Resistance Training with Wearable Sensors

**DOI:** 10.3390/s25092662

**Published:** 2025-04-23

**Authors:** Cheuk-Yan Au, Chee Ming Noel Sng, Jeshuan Heng, Thanh Tam Nguyen, Joo Chuan Yeo, Ali Asgar Saleem Bhagat

**Affiliations:** 1 Institute for Health Innovation & Technology (iHealthtech), National University of Singapore (NUS), MD6, 14 Medical Drive, #14-01, Singapore 117599, Singapore; cy.au@nus.edu.sg (C.-Y.A.); jcyeo@microtube.tech (J.C.Y.); 2 Department of Biomedical Engineering, National University of Singapore (NUS), 4 Engineering Drive 3, Singapore 117583, Singapore

**Keywords:** gamified exercise, wearable sensors, strength adaptation, exergames, resistance training

## Abstract

Gamification has been extensively applied to aerobic and cardiovascular training, but its adoption in resistance training remains limited. Unlike traditional strength training, which often relies on intrinsic motivation and discipline, gamification introduces extrinsic incentives and real-time feedback that improves engagement and adherence, affecting overall training outcomes. In this work, we develop a gamified resistance training program using wearable sensors to explore the potential benefits of interactive and data-driven exercise experiences. By leveraging real-time feedback and performance tracking, our system provides participants feedback to properly control key training variables such as form and tempo, essential factors for muscle hypertrophy and strength development. To evaluate the effectiveness of our approach, we conducted a short-term comparative study in which participants were assigned to either a gamified training group or a conventional resistance training control group. Over a four-week period, we assessed volitional adherence to prescribed tempo and repetition schemes, along with strength adaptations in the biceps and triceps. Our findings indicate that gamified resistance training significantly enhances adherence to tempo and repetition targets while fostering better adaptation to the workout regime. Participants in the gamified group exhibited measurable improvements in upper body strength compared to the control group. These results suggest that gamification when integrated with wearable sensor technology, can be a powerful tool for optimising resistance training effectiveness and motivation.

## 1. Introduction

Gamification has emerged as a powerful strategy to enhance motivation, engagement, and adherence in various domains, including education, healthcare, and fitness [1]. In the context of exercise, gamification involves incorporating game-like elements such as challenges, rewards, leader-boards, and real-time feedback to make exercises more engaging and enjoyable [2]. Traditional exercises often struggle with long-term adherence due to a lack of motivation and other personal obligations [3]. By leveraging gamification, fitness interventions can provide extrinsic motivators that sustain participation, ultimately leading to better health outcomes [4]. Research has shown that adding elements of play and competition to exercise programs can increase user retention, improve self-efficacy, and encourage consistent physical activities [5].

Most gamified fitness applications and interventions strongly focus on aerobic and cardiovascular training, leveraging the accessibility and convenience to perform these exercises on demand [6]. Activities such as running, cycling, and high-intensity interval training (HIIT) have been extensively gamified using wearable technology, mobile applications, and virtual environments. These applications often incorporate leaderboards, virtual rewards, and real-time feedback to encourage user engagement and adherence [7]. Fitness games such as “Zombies, Run!” and “Beat Saber” have successfully integrated immersive game mechanics to increase motivation for aerobic exercise [8,9]. Wearable fitness trackers and smartwatches further enhance engagement by providing continuous monitoring of heart rate, calories burned, and distance travelled, reinforcing positive behaviors through immediate feedback [10]. Despite the success of gamification in aerobic and cardiovascular fitness, its application in resistance training has received comparatively little attention, even though strength training is a crucial component of overall fitness and health.

In the past 10 years, researchers and developers have begun to explore the potential of gamifying resistance training, recognizing its role in improving muscle strength, endurance, and overall physical health [11]. Unlike aerobic exercise, resistance training involves a complex program consisting of exercise selection and order, varying levels of resistance to maintain exercise intensity, and a strong focus on the precise execution of specific techniques during the workout regime [12]. There were two notable attempts to gamify strength training: StrengthGaming, a Flappy Bird inspired game using now discontinued wearable EMG sensors [MYO armband, Thalmic Labs] [13] to track and control a virtual character using rhythmic repetitions in free weight training, and Exermon [14], a Tamagotchi-style breeding game in which the avatar monster is fed and gains stats when the player is performing bodyweight exercises using the accelerometer on a smartphone. No commercially available wearables or exergames made specifically for resistance training were found. Key metrics in resistance training include repetition count, time under tension (TUT), movement velocity, and muscle activation patterns, all of which are critical for optimising strength development and hypertrophy [15]. Proper adherence to form, tempo, and rest intervals significantly impacts training outcomes, making real-time feedback a valuable tool in ensuring effective workouts [16]. Given the structured nature of resistance training, gamification can play a crucial role in reinforcing adherence to correct techniques and training principles, ultimately enhancing both safety and effectiveness.

Wearable sensors have been instrumental in the rise of gamified fitness, providing real-time data on various physiological and biomechanical parameters. In aerobic and cardiovascular fitness, wearable devices such as smartwatches and chest straps monitor heart rate, steps, and calories burned, offering users continuous feedback and performance tracking [17]. In the context of resistance training, inertial measurement units (IMUs) have been widely used to track movement patterns, repetition counts, and lifting velocity [18]. IMUs, which consist of a 3-axis accelerometer and a 3-axis gyroscope, can be embedded in wearables such as smartwatches, fitness bands, or even directly in resistance training equipment to capture detailed motion data [19]. Several gaps remain in the current application of wearable sensors in resistance training, most gamified fitness solutions focus on basic repetition. They are also incapable of capturing muscle dynamics restricting their effectiveness in providing comprehensive biomechanical feedback [20] and other training variables such as tempo, time under tension, or form adherence. Furthermore, the integration of wearable sensors into immersive and interactive gamified experiences specifically for resistance training is still in its infancy [21]. Without comprehensive systems that leverage both real-time biomechanical data and game mechanics, the potential benefits of gamified resistance training remain largely untapped.

This work aims to explore the gap in gamified resistance training by developing an interactive workout regime program that leverages wearable sensors to measure key training parameters to provide real-time feedback via gameplay. With this idea, we developed a sensing unit and a series of games that focused on controlling essential resistance training variables such as form and tempo that directly impact the engagement of the muscles, affecting strength adaptation [22]. The unit allows for the capability to measure muscle expansion of the targeted muscle as a proxy to muscle effort and enables the detection of exercises that could potentially be obscured from cameras or isometric exercises with no joint movements. We conducted a comparative study assessing the effectiveness of gamified resistance training versus conventional training in terms of adherence, repetition uniformity, and strength gains in the biceps and triceps over a four-week period. Our findings demonstrate that gamified resistance training not only improves user engagement but also facilitates better adherence to structured workout protocols, leading to measurable improvements in strength. Through this work, we contribute to the growing field of gamified fitness by providing a structured and evidence-based approach to integrating gamification into resistance training using wearable technology.

## 2. Materials and Methods

### 2.1. Smart Sensing Unit

**Figure 1:**
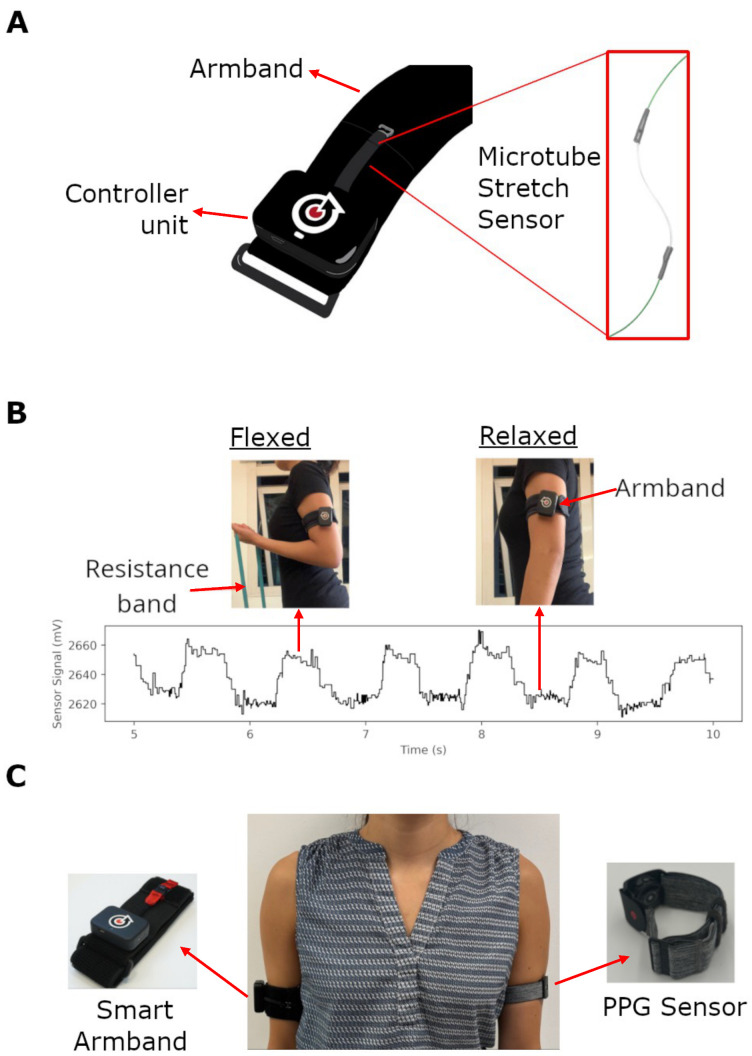
(**A**) The smart textile armband consists of a controller unit and an embedded microtube stretch sensor (Right); (**B**) Changes in smart armband sensor signal reflecting the expansion in upper arm circumference when flexing under load from a resistance band; (**C**) A participant wearing the smart armband on her dominant arm across the medial compartment of their biceps and triceps, as well as the PPG sensor on the other arm.

The modular smart sensing unit consists of a flexible microtube stretch sensor [23] and a controller unit (Figure 1A). The microtube stretch sensor is created by injecting a liquid metal alloy (eutectic Gallium Indium, or eGaIn) into a 5 cm long silicone microtube [24], which is plugged at both ends with thin copper wires and sealed with epoxy resin. When the sensor is deformed, this results in a change in resistance, which is reflected as a change in the electrical signal passing through the sensor. The wires are then soldered directly onto an adapted controller unit from our previous work [25], which consists of a microcontroller (MCU), a Bluetooth Low-Energy (BLE) transmitter, an inertial measurement unit (IMU), and a 24-bit analogue-to-digital converter (ADC) to acquire and transmit the sensor signals via Bluetooth Low Energy (BLE) at 60 Hz [RS Components Singapore]. Upper arm exercises are a popular form of free weight strength training. Specifically, the elbow joint was chosen as it is a hinged, single-jointed movement which could be easily be isolated for measurement, as well as training [26]. For the purpose of this study, the sensing unit was translated into a smart armband using an off-the-shelf flexible textile armband that could accommodate upper arm size from 22 cm to 34 cm in circumference. Modifications include adding a loop of Velcro to mount the controller unit and encasing the microtube stretch sensors in a fabric sleeve with a hook at the latter end to attach it to the armband. The armband has an adjustable loop closure to fit multiple physiques and when worn snugly, the smart armband tracks changes in the upper arm’s circumference during various movements, including concentric elbow flexion and extension (primarily controlled by the biceps and triceps, respectively) and isometric contractions, with no visible movement of the elbow occurring. To ensure accurate measurements, the smart armband needs to be calibrated before each session. The calibration process sets threshold limits by measuring the sensor’s signal in two distinct states: the relaxed state and the flexed state during flexion. The lower bound of the threshold is determined from the signal in the relaxed state, while the upper bound comes from the signal in the fully flexed state (Figure 1B). Since this calibration applies to all exercises, each game includes a threshold toggle for fine-tuning as needed. These two thresholds then define the signal range specific to each user, ensuring consistency and reliability in data acquisition.

### 2.2. Experiment Protocol

A structured experimental protocol was developed to investigate the effects of gamification on upper-limb resistance training, specifically targeting the biceps and triceps, through the use of resistance bands. The study employed a smart armband to monitor participant performance and engagement. Eligible participants were healthy adults aged 21 to 60 years who demonstrated the ability to comprehend and follow study instructions. Inclusion criteria required that participants had no pre-existing cardiovascular conditions, no current orthopaedic injuries, and no other non-injury-related conditions that could impede participation in resistance training. Furthermore, participants were required to have been physically inactive, defined as failing to meet the recommended moderate physical activity level of at least 150 min per week, for at least three months prior to enrolment. Participants were excluded if they had sensitive skin, open wounds, or rashes on the upper arm, as these conditions could interfere with the proper use of the smart armband. Additionally, individuals with a history of motion sickness triggered by video game exposure were excluded to prevent potential discomfort during gamified training sessions. This study employs a two-arm parallel-group design, consisting of a control group and a game group. Participants in the control group perform the prescribed resistance exercises under a one-to-one direct supervision and instruction of the study coordinator in a closed-door setting. In contrast, participants in the game group are guided by an interactive game designed to facilitate exercise engagement. To ensure consistency, both groups perform identical exercises for each session. The entire study consists of a total of 12 sessions conducted over four weeks, with a frequency of three sessions per week. All sessions take place in a designated room at the university, equipped with a screen for exercise guidance and gamification elements. To ensure participant compliance and minimize attrition, a monetary incentive is provided upon study completion.

### 2.3. Exercise Plan and Exergame Design

Upon arrival at each session, participants were instructed to wear the smart armband on their dominant arm, positioned across the medial compartment of the biceps and triceps. To monitor heart rate during the session, an off-the-shelf photoplethysmography (PPG) sensor [Verity Sense, Polar Technology Co., Ltd., Kempele, Finland] (Figure 1C) was placed on the non-dominant upper arm. Muscle strength assessments were conducted using a digital handheld dynamometer [MicroFET 2, Hoggan Scientific, Salt Lake City, UT, USA] to measure peak biceps and triceps forces. Measurements were performed following the manufacturer’s standardized protocol for elbow flexion and extension. Concurrently, the smart armband was calibrated by recording baseline signals while the participant was in a relaxed state. The maximum signal values were determined during elbow flexion and extension tests using the dynamometer, establishing reference points for subsequent exercise data collection. Prior to engaging in the prescribed workout regimen, all participants completed a standardized full-body warm-up, guided by an instructional video [27], to ensure muscle preparedness and reduce the risk of injury. The workout plan consisted of three unique exercise series, each comprising four strength training exercises (Table 1). Each session included two concentric exercises targeting the biceps, one isometric exercise, and one eccentric exercise for the triceps. The participants performed the exercise series based on the session number they were attending, e.g., the participant performed series A on their 1st, 4th, 7th and 10th session. This structure was designed to introduce variation and maintain participant engagement throughout the study. Despite the variation, the number of sets and repetitions, as well as the tempo remains the same for the exercises. The tempo is the pacing for each repetition and it has 4 numbers which refer to: time for concentric or eccentric movement from resting position, time staying under tension, time to return to resting position, and time staying at a resting position, respectively. The three sessions were repeated over four weeks, resulting in a total of 12 workout sessions. During each session, participants used a standardized 15 kg resistance band [Corength, Decathlon SA, Lille, France] (Figure 1B) for all workout regimes. To ensure adequate recovery and preparation, a 1-minute rest interval was provided between exercises. During this period, all participants received instructions for the subsequent exercise. At the conclusion of each session, post-exercise muscle strength assessments were conducted using the digital handheld dynamometer to measure elbow flexion and extension forces, enabling comparison with pre-exercise values.

**Figure 2:**
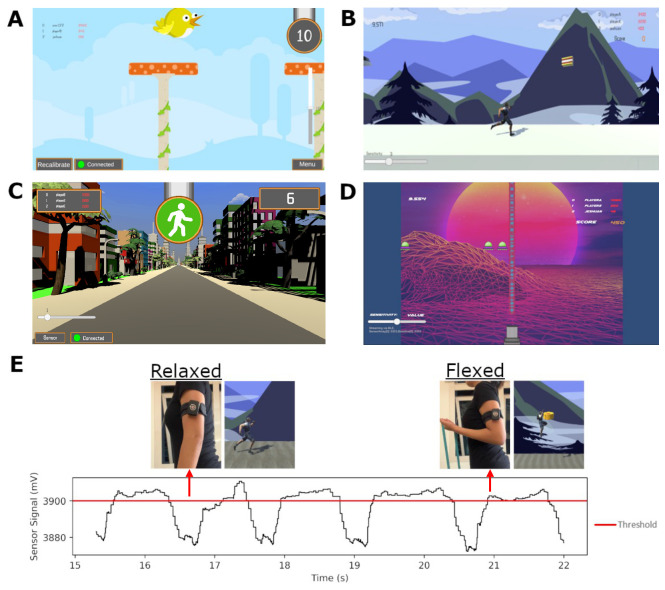
Four exergames were developed for this study specifically for the target exercises, each with a distinct theme: (**A**) Flutter Fly; (**B**) Mountain Dash; (**C**) Signal Sprint; (**D**) Alien Defence; (**E**) Demonstration of how the smart textile band functions with Mountain Dash: the avatar jumps to collect the coins when the participant flexes, a resistance band was used to attain the required training intensity. The threshold is set to 90%.

A custom software program was developed using Unity 2021.3 to integrate seamlessly with the smart armband, enabling both data collection and interactive exergaming. This program facilitated the export of raw sensor data from the smart armband for further analysis and served as the primary interface for the four exergames (Figure 2A–D) specifically designed for this study. These exergames with distinct themes featured customisable interval settings, were developed to correspond directly with the exercises in the workout plan (Table 1), allowing for a structured comparison between the control and game groups. Both groups utilised the program for smart armband calibration and sensor data export. However, only the game group engaged with the exergames. Additionally, an on-screen slider was included to adjust the activation threshold required for gameplay (Figure 2E) when needed. By default, this threshold was set to the median value within the participant’s calibration range.

The four exergames were designed to incorporate the targeted movements and muscle activations of the prescribed exercises:

1. Flutter Fly—The participant flexes and relaxes across the set threshold to control a bird avatar’s vertical movement, collecting gems while avoiding obstacles.
2. Mountain Dash—A side-scrolling coin collection game in which the participant flexes above the threshold to make the avatar jump and collect coins at designated intervals.
3. Signal Sprint—The avatar continuously walks forward, and the participant must flex and hold above the threshold to stop movement. The game features a randomized green light (walk) and red light (stop) phase.
4. Alien Defence—A side-scrolling shooter game where the participant flexes to fire a laser. The laser can be activated continuously by maintaining a flexed posture. Enemies appear randomly from the left side of the screen.

After completing the standardized warm-up, control group participants were shown each exercise and received instructions on repetition count, tempo, and rest intervals before beginning. In contrast, game group participants were instructed on gameplay mechanics and given the opportunity to adjust their smart armband thresholds for optimal responsiveness. Throughout the session, the study coordinator was responsible solely for timing rest intervals for both groups, ensuring consistency in exercise pacing and recovery periods.

## 3. Results

A total of six participants (four males, two females) with a mean age of 21.7 ± 1.2 years were successfully recruited for the study. Participants were briefed on the experimental protocol, provided with a written copy for reference, and signed an informed consent form in person. To ensure eligibility, they were verbally screened against the inclusion criteria. A monetary incentive was offered upon successful completion of the study to encourage adherence. Recruitment was conducted internally within the National University of Singapore (NUS) through circular email announcements. Participants were alternately assigned to one of two study groups—control or game—based on the order of recruitment, beginning with the control group. Each participant was assigned a unique three-character alphanumeric identifier, where ‘C’ represented the control group and ‘G’ represented the game group, followed by a sequential two-digit number based on recruitment order (e.g., C03, G01). Participants were scheduled for three sessions per week, totalling 12 sessions over the course of the study. All participants successfully completed all 12 sessions within an average duration of 30 ± 1.55 days. Collected data were anonymized and accessible only to authorized research personnel. The study commenced in February 2023 and concluded in October 2023. All data processing was conducted using Python 3.9.13 [28].

Heart rate data were continuously recorded at a 1 Hz sampling rate using the Polar Android application and exported in CSV format at the end of each session. No additional pre-processing was performed on the raw data. To facilitate analysis across participants of varying ages, heart rate values were normalized as a percentage of maximum heart rate (*%maxHR*) using the following equation [29]:

(1) $$\%maxHR=\frac{Heart\;Rate\;(BPM)}{220-Age}$$

**Figure 3:**
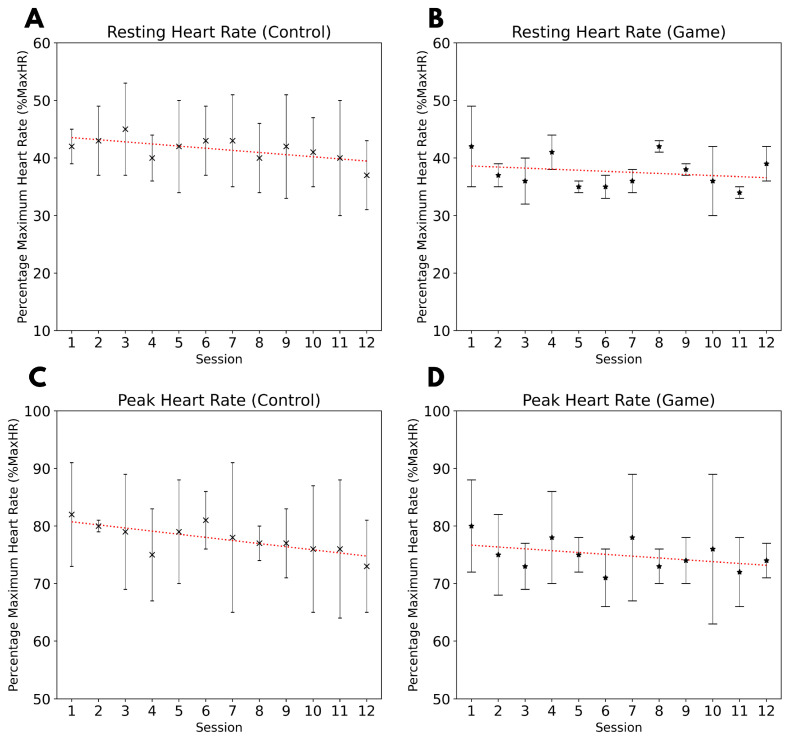
A slight declining trend (indicated by red dotted line) in resting and peak heart rate as a percentage of maxHR was observed through the 12 training sessions. Resting heart rate (*%maxHR*) was measured before the start of each workout session for the control group (**A**) and the game group (**B**). Peak heart rate (*%maxHR*) was measured during each workout session for the control group (**C**) and the game group (**D**).

For each participant, resting heart rate and peak heart rate during exercise were extracted. At the start of each session, both groups exhibited an average resting heart rate of approximately 40% maxHR (Figure 3A,B). During exercise, participants reached an average peak heart rate of approximately 75% maxHR (Figure 3C,D), indicating that the prescribed workout regime effectively induced a moderate to vigorous intensity level. Additionally, a mild declining trend in *%maxHR* was observed over the training period, suggesting physiological adaptation to the workout regime.

**Figure 4:**
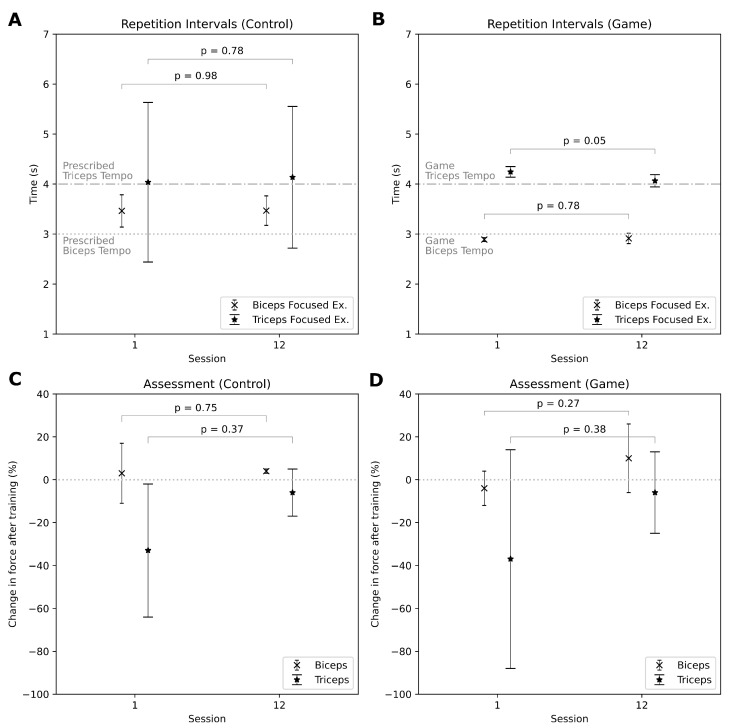
(**A**) Mean and standard deviation of repetition intervals between the first and last session for the control group’s biceps-focused exercises (Exercise 1 and 2) and triceps-focused exercise (Exercise 4). The data was analyzed via a two-tailed *t*-test; the *p*-value shows significant difference in repetition interval. The prescribed tempo for the respective muscle groups highlights adherence by the participants, and the *p*-value shows if there are statistical significant changes by the participants over the sessions; (**B**) Mean and standard deviation of repetition intervals between the first and last session for the game group’s biceps-focused exercises (Exercise 1 and 2) and triceps-focused exercise (Exercise 4). The game group shows better adherence than the control group by being closer to the prescribed tempo, as well as lesser variation. This group also shifted significantly towards the prescribed tempo after 12 sessions; (**C**) Percentage change in force of assessment measured by the dynamometer between the first and last session for both biceps and triceps in the control group. The grey line at 0% reduction in force indicate that the participant was not fatigued post-session. The *p*-value shows statistical significance in difference between them; (**D**) Percentage change in force of assessment measured by the dynamometer between the first and last session for both biceps and triceps in the game group. While both groups seen a lower reduction in triceps force which indicated adaptation to the exercises, increase in variation in the game group for both biceps and triceps was observed.

The microtube stretch sensor embedded within the smart armband (Figure 1A, in red) was designed to detect muscle expansion and contraction in the upper arm during exercises. The raw sensor signals were sampled at 60 Hz and stored in CSV format for subsequent analysis. In the game group, these signals were also used as real-time input for the exergames. The raw signals were filtered using a third-order Butterworth bandpass filter (0.1–1 Hz) to reduce motion noise while preserving the signal profile for concentric exercises (Exercise 1, 2, and 4). No filtering was performed on the isometric contraction (Exercise 3) to retain the profile of sustained muscle activation patterns. The processed signals were normalized using the calibration baseline (relaxed state) and the peak contraction value. The mean and standard deviation of the repetition intervals were calculated for concentric biceps exercises (Exercise 1 and 2) and tricep exercises (Exercise 4) by performing peak-to-peak analysis for Exercise 1, 2, and Exercise 4, respectively (Table 2). The mean of the repetition interval represents the participant’s adherence to the prescribed tempo, while the standard deviation (SD) of these intervals reflects uniformity in execution. For the concentric biceps exercises (Figure 4A,B), the mean repetition intervals in the first session were (Control 3.46 s ± 0.32, Game: 2.89 s ± 0.04) and after 12 sessions (Control 3.47 s ± 0.29, Game: 2.91 s ± 0.1), with the prescribed tempo being 3 s per repetition. The game group (Figure 4B) has better uniformity in executing the biceps exercises seen from the low SD values, no significant differences were found between the first and last sessions in either group. For triceps exercises, a significant difference was observed in the game group between the first session (Control 4.04 s ± 1.6, Game: 4.24 s ± 0.10) and the last session (Control 4.13 s ± 1.42, Game: 4.06 s ± 0.12). Once again the game group exhibited better uniformity, they were also able to bring the mean repetition interval extremely close to the prescribed tempo of 4 s per repetition after 12 sessions.

The peak biceps and triceps forces (N) were assessed using a digital handheld dynamometer, adhering to the manufacturer’s protocol for elbow flexion and extension. Measurements were obtained pre- and post-exercise for each session and logged into a spreadsheet. No additional pre-processing was required. Addressing inter-individual variability, normalization was achieved by calculating the intra-session change in muscle force, facilitating direct comparisons across participants. During the first session, participants in both groups exhibited minimum difference in biceps force after completing the workout regime (Control: 3±14%, Game: −4±8%). After 12 sessions of training, the control group (Figure 4C) was able to maintain similar force levels pre- and post-exercise, while the game group (Figure 4D) demonstrated a nominal increase in biceps force post-exercise (Control: 4±1%, Game: 10±16%), indicating potential improvements in muscular endurance. For triceps force, both groups exhibited a significant decline in force after exercise during the first session (Control: −33±31%, Game: −37±51%). However, after 12 sessions, the post-exercise force reduction became minimal (Control: −6±11%, Game: −6±19%) suggesting improved muscular resilience over time. Despite these observed trends, none of the differences reached statistical significance indicating that while training may have led to slight improvements in force retention, the effects were not pronounced within the study duration.

## 4. Discussion

Repetition maximum [16] is an important metric used in strength training and exercise to determine the appropriate resistance level for an individual. By identifying the maximum weight or resistance a person can handle for a specific number of repetitions, we can tailor workouts to optimise performance and prevent injury. While repetition maximum (RM) would typically be the guiding factor in selecting resistance, in this work a 15 kg resistance band was standardized among the participants instead of being influenced by several practical considerations. Firstly, there are four different workout regimes performed in each session; the variability makes it difficult to determine an ideal resistance level based on the RM of the participants’ biceps and triceps. Additionally, the short rest periods between sets require participants to sustain their effort without excessive fatigue, making it essential to use a resistance that does not push them to their absolute limits. Another key reason for selecting a 15 kg resistance band was to ensure that users could maintain proper form throughout the exercises. When resistance levels are too high, participants may struggle to execute movements correctly, leading to potential injuries or reduced effectiveness of the workout. By opting for a resistance level well below each individual’s 1RM, the study prioritises the quality of every repetition over maximum strength output, which is especially important in a dynamic training environment [30].

This study incorporated continuous heart rate monitoring using an upper-arm photoplethysmography (PPG) sensor to ensure participants maintained an appropriate heart rate range during exercise. Monitoring heart rate is essential for optimizing training benefits while minimizing the risk of overexertion. By maintaining participants within the moderate to vigorous heart rate training zone, the study aimed to enhance endurance and overall fitness without imposing excessive cardiovascular strain [18]. The selection of an upper-arm PPG sensor was intentional, prioritizing both wearability and measurement accuracy. While a wrist-based PPG device was initially considered, it was excluded due to motion artifacts caused by arm movements. A chest strap was also rejected, as it posed potential comfort and accessibility concerns, particularly for female participants. Across both the control and game groups, participants’ heart rates increased from approximately 40% of their maximum heart rate at rest to around 75% during exercise, confirming that the prescribed workout regime (Table 1) effectively elicited moderate to vigorous exercise intensity [31,32]. Furthermore, a gradual decline in heart rate responses over the four-week training period was observed in both groups, indicating physiological adaptation to the workout regime [33].

A key advantage of the smart armband lies in its integration of a microtube stretch sensor, which enabled direct measurement of muscle expansion during exercise. Although inertial measurement units (IMUs) and computer vision technologies are effective in tracking motion, they are incapable of capturing muscle deformation, a crucial parameter for evaluating force exertion and muscle engagement. This limitation highlights the need for a flexible force sensor, such as the microtube stretch sensor, which can be positioned around the muscle group to provide real-time, direct measurements of muscle expansion for isometric and concentric muscle contractions. This capability provides a more comprehensive evaluation of training effectiveness. However, due to the nature of the sensor, electromyography (EMG) normalization methods such as Maximum Voluntary Contraction (MVC) as done using the handheld dynamometer, were deemed unsuitable for intersubject normalization of the amplitude of the smart armband as inter-individual differences in muscle expansion, due to variations in body composition, were independent from the force output. Consequently, this study could not derive real-time estimations of force output using the smart armband. Analysing the mean values for exercise tempo adherence between the control group and the game group, both groups demonstrated performance uniform with the prescribed tempo. For biceps-focused exercises, the control group exhibited a steady mean repetition time of 3.4 s, with a slight reduction in standard deviation (SD) from 0.32 to 0.29 after 12 sessions. The game group maintained a uniform mean repetition time slightly below the prescribed tempo at 2.9 s, likely due to anticipatory behavior associated with gem collection tasks from the exergames with exceptionally low SD values (Session 1: 0.03, Session 12: 0.1). It suggested the 15kg resistance band did not induce fatigue on biceps within a session for either group. The control group, lacking exergame guidance, demonstrated wider SD values, though no fatigue-related trends were detected. For triceps-focused exercises, a notable discrepancy was observed between the groups. The control group exhibited performance less uniform than the control group, as evident by higher SD values, although a slight reduction in variability was noted after 12 sessions. Conversely, the game group displayed uniform performance throughout. This disparity implied that the 15 kg resistance band imposed fatigue on participants’ triceps muscles, yet the game group benefitted from the exergame mechanics reinforcing performance uniformity. In contrast, the control group, lacking in mechanisms providing immediate feedback struggled to maintain uniformity. Both groups’ mean repetition times remained close to the prescribed tempo of 4 s throughout the sessions. This suggests that gamified workouts can enhance the uniform performance of resistance training. These micro parameters could potentially make a difference in the training outcome.

The results obtained from the handheld dynamometer provide valuable insights into the strength adaptations of the biceps and triceps over the 12-session training period. A significant initial decline in triceps strength was observed immediately after the first session in both the control and game groups (Figure 4C,D). This decline is likely due to muscle fatigue resulting from unaccustomed resistance training, particularly given that participants were requested to stop exercise three months prior to joining the study. Additionally, the triceps muscle is naturally weaker than the biceps, making it more susceptible to early fatigue. However, over the course of the study, a notable adaptation was observed, with a reduction in the percentage change in triceps strength before and after each session, suggesting improved muscular endurance by the end of the 12th session. In contrast, biceps strength did not exhibit a significant decline following the first session, indicating that the 15 kg resistance band may not have provided sufficient load to induce immediate fatigue in the biceps. Interestingly, by the end of the 12-session intervention, a minor increase in biceps strength was observed in the game group but not in the control group. One possible explanation is that the game mechanics enforced stricter adherence to tempo, leading to more controlled and uniform repetitions, which may have positively influenced strength development, as seen with higher mean and variability in the game group after 12 sessions. However, further research is necessary to confirm this effect and determine whether tempo regulation in gamified workouts plays a significant role in strength adaptation. Beyond strength measures, progressive adaptation was also evident in heart rate data, reinforcing the idea that repeated exposure to resistance exercise leads to improved muscular and cardiovascular efficiency. This trend was uniform across both the control and game groups, suggesting that training modality was not a limiting factor in achieving adaptation. These findings align with prior research indicating that early strength declines do not necessarily reflect a lack of progress, but rather an expected physiological response to new physical demands, particularly in untrained individuals.

Overall, the results emphasize the importance of structured resistance training in facilitating progressive muscular adaptations. While gamified training appears to encourage greater uniformity in movement execution, further studies are needed to explore long-term effects and whether progressive overload or increased resistance in exergames could yield greater strength gains. Future work should also investigate whether individualized game difficulty settings could further enhance training effectiveness and engagement.

## 5. Conclusions

This study demonstrates the potential of gamification in strength training through the integration of wearable sensor technology and interactive exergames. Our findings suggest that gamifying resistance training workout regimes promote uniformity in performance compared to traditional, volitional workouts. The structured nature of exergames complements the predefined movement patterns and tempos, contributing to the reduced variability in the performance of repetitions as evident from the lower standard deviation observed in the game group. Despite these promising findings, no statistically significant differences were observed in muscle strength gains or heart rate responses between the game and control groups after four weeks of training. Physiological impacts may require longer intervention period or progressive overload strategies to elicit measurable improvements in muscular strength and cardiovascular endurance.

Future research should explore extended training durations, increased exercise intensity progression, and the incorporation of a wider variety of game mechanics to further engage participants and enhance training outcomes. Additionally, refining real-time feedback mechanisms and personalising game difficulty based on individual performance metrics may optimise motivation, adherence, as well as understanding the implications of uniform repetition performance. Investigating the effects of gamification on different populations, such as older adults or individuals undergoing rehabilitation, could also provide valuable insights into its broader applications in fitness and healthcare, paving the way for future advancements in interactive fitness technologies. 

## Figures and Tables

**Table 1:** Structured workout plan detailing the workout regime performed, tempo, and the corresponding games. A total of 3 exercise series were designed; Exercises 1 and 2 are concentric for the biceps, Exercise 3 is an isometric static hold targeting both the biceps and triceps brachii, and Exercise 4 is an eccentric exercise for the triceps. Tempo is the pacing for each repetition and it has 4 numbers which refer to: time for concentric or eccentric movement from a resting position, time staying under tension, time to return to resting position, and time staying at resting position, respectively. The participants performed the plan according to the session number for a total of 12 sessions, performing each series four times in total.

	Series A	Series B	Series C	Reps × Sets	Tempo (s)	Game Name
Session No.	1/4/7/10	2/5/8/11	3/6/9/12			
Exercise 1	Bent over rows	Upright row	Seated row	12 × 3	1/0/1/1	Flutter Fly
Exercise 2	Lunges with biceps curl	Squat to biceps curl	Biceps curl	12 × 3	1/0/1/1	Mountain Dash
Exercise 3	Hammer curl static hold	Seated row static hold	Biceps curl static hold	1 × 6	1/10/1/10	Signal Sprint
Exercise 4	Overhead triceps extension	Squat shoulder overhead press	Squat shoulder overhead press	12 × 3	2/0/1/1	Alien Defence

**Table 2:** (**A**) Summary of results across repetition intervals, comparing mean values and variability against prescribed and game tempos; (**B**) Percentage change in force as measured by a dynamometer, highlighting immediate fatigue effects of the exercise session.

Summary of Results					
A. Repetition Intervals (s)					
Muscle Group	Prescribed/ Game Tempo	Control Group		Game Group	
		Session 1	Session 12	Session 1	Session 12
Biceps	3	3.46±0.32	3.47±0.29	2.89±0.04	2.91±0.1
Triceps	4	4.04±1.6	4.24±0.10	4.13±1.42	4.06±0.12
B. Dynamometer Assessment (% change in force)					
Muscle Group		Control Group		Game Group	
		Session 1	Session 12	Session 1	Session 12
Biceps		3±14	4±1	−4±8	10±16
Triceps		33±31	−37±51	−6±11	−6±19

## Data Availability

The data presented in this study are available on request from the corresponding author.
